# Virtue of courage as modulator in interactions among oral health beliefs, oral hygiene habits and dietary preferences

**DOI:** 10.6026/973206300200649

**Published:** 2024-06-30

**Authors:** S Supriya, Rajbir Singh, Amra Ahsan

**Affiliations:** 1 Faculty of Behavioral Sciences, SGT University, Gurugram, Haryana, India

## Abstract

The impact of virtue of courage as mediating and moderating variable in interactions of oral health beliefs with oral hygiene habits and dietary preferences is of interest. A total of 100 patients with a complain of periodontal ailments and dental caries were enrolled from dental institute. 40 items belonging to character strengths of psychological virtue of courage in values in action Inventory of Strength (VIA-IS) Questionnaire and 15 Oral Health Belief Questionnaire items were recorded. Oral hygiene habits and dietary preferences congruent with oral health were also recorded. A significant positive correlation was observed in the following variables: barriers/gender (correlation coefficient =.212, P =.034) There was positive correlation between perseverance and nutritional preferences (correlation coefficient = .239, P =.017); courage and nutritional preferences (correlation coefficient = .241, P = .016). Virtue of courage and its character strengths have significant positive impact over dietary preferences congruent with oral health. Inculcation of character strengths belonging to courage as virtue may have the potential to modulate compliance to oral health beliefs and consequently oral health.

## Background:

High prevalence of non-communicable diseases with significant impact of associated morbidity and mortality inspite of availability of health care facilities and services hints towards the potential role of probable barriers to seeking health care services and/or to adopting recommended preventive measures for management of associated modifiable risk factors. Oral diseases, the most widespread diseases of humanity caused by modifiable risk factors, affect people throughout their lifetime [1]. Oral health problems include, but not restricted to, pain, halitosis, compromised mastication efficiency, drifting of teeth disfigurement and impaired esthetics. Furthermore some of these may act as chronic stressors and can have impact on the person's psychological, social and physical functioning. Oral diseases affect close to four billion people worldwide [2]. The influence of dental hygiene is not restricted to oral cavity only. Microbial constituents of dental plaque have been observed at distant vital organs including heart and brain [3]. These findings have been linked to systemic implications associated with oral pathogens [4]. Perusal of literature reveals significant emphasis being placed on the relevance of widespread prevalence of emotion of dental fear and anxiety with reference to oral health behaviours and utilization of oral health facilities [5]. Various scales have been designed and validated for its assessment. Pharmacological management and psychological strategies are being discussed. Perspective on human behaviour under investigation should not be restricted to psychological weaknesses. Positive psychology stresses on balanced view of individual appraisal [6]. A comprehensive individual appraisal about strengths and weaknesses along with resources and ecological stressors may be effective in predicting and modifying health outcomes and functioning. Freedom from disabling anxiety is one of the important characteristics of mental health. Psychological ingredients of character strengths related to courage as virtue may be a prerequisite for its achievement. The concept of applications of courage is a routinely required phenomenon in our day-to-day activities. It helps to face life challenges. Perseverance in the face of fear and anxiety, particularly during disease, is a manifestation of courage. Compliance to therapeutic recommendations is believed to be poor with chronic diseases, particularly those perceived to be non-threatening [7]. Non-compliance or erratic compliance in health behaviours is linked to fear, stressful events of life and the associated health beliefs. Resistance against surrendering to temptations or illnesses may be acquired through discovering our character strengths related to virtue of courage viz. bravery, perseverance, integrity and zest. Discovering, inculcating and utilising these character strengths, particularly during periods of deviations from health to pathology may prove to assist healing and improve health outcomes. Relevance of individual responsibility in maintaining health and preventing diseases and disorders by self-initiated activities cannot be ignored. Health belief model (HBM) is being employed successfully across disciplines to explain and predict health related behaviours [8,9]. However, factors modulating health beliefs and their role as mediating/ moderating variables needs to be investigated further. Perceived barriers in HBM refer to potential negative aspects of a particular health action acting as impediments for the recommended health behaviours. Individual perception of pain and adverse feelings associated with invasive nature of dentistry may comprise important barrier in primary and secondary preventive oral health seeking initiatives. Among the positive psychological interventions, relevance of psychological virtue of courage in modulating health behaviours has been suggested. It has been conceptualized as positive behavioural approach amidst emotions of fear [10,11]. It acts as a deterrent against development of pathological anxiety [12]. Therefore, it is of interest to document the impact of virtue of courage as mediating and moderating variable in interactions of oral health beliefs with oral hygiene habits and dietary preferences.

## Materials and Methods:

**Figure 1:**
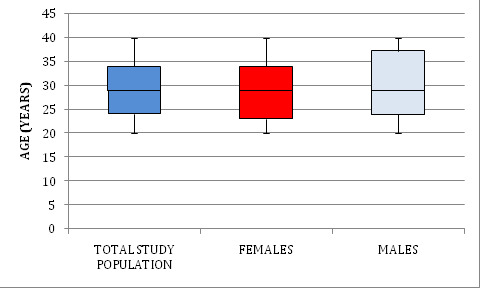
Box-and- whisker plot depicting age (years) in total study population, females and males. The median (minimum; maximum) age (years) in study population was 29.000(20.00;40.00). Females and males had median (minimum; maximum) age of 29.00(20.00; 40.00) and 29.000(20.00;40.00) respectively.

100 patients with a chief complaint with reference to periodontal ailments and dental caries were recruited from dental institute (Figure 1). Written informed consent was obtained before the enrolment. The study protocol was approved by the Ethics Committee of Faculty of Behavioural Sciences, SGT University, and Gurugram. The study was carried out as per ethical principles of Declaration of Helsinki 1975, as revised in 2013.

## Tools:

## Values in action inventory of strength (via-is) questionnaire:

VIA-IS was developed by Peterson and Seligman (2004) [13]. It consists of 240 items measuring 24 character strengths. Participants are required to answer each item on the Likert scale ranging from 1 (very much unlike me) to 5 (very much like me). As the scope of study was with reference to character strengths related to virtue of courage, score of 40 items, 10 each of the four associated chacter strengths that constitute the courage were taken into consideration in the present study. Four character strengths comprising the virtue of courage are: Bravery (valour) Persistence (perseverance, industriousness) Integrity (authenticity, honesty) Vitality (zest, enthusiasm, vigour).

## Oral health belief questionnaire:

This 18 oral health belief items based questionnaire has been found to possess good reliability and validity among diverse populations. [14] Belief items measure various dimensions of the health belief model such as perceived seriousness of oral diseases, perceived importance of oral health, perceived barriers to oral health care, benefits of plaque control and efficacy of dentists. 15items related to theoretical construct of perceived seriousness (4), barriers (2), motivation (1), perceived benefits of prevention and plaque control (6) and efficacy of dentist (2) were considered in this study. Items with reference to perceived importance (3) are not a part of this paper.

## Statistical analysis:

Statistical analysis was performed using the statistical software program SPSS (v. 20, IBM). Normality of data was determined using the Shapiro-Wilk test. Data are presented as mean ± standard deviation (SD) and median (25th percentile-75th percentile). Difference in between groups was assessed using the unpaired student t test (for normally distributed data) and Mann-Whitney U test (for no normally distributed data). Gender, being a categorical variable is presented as number (percentage). Spearman rank correlation coefficient was calculated to determine the association between two non-normally distributed continuous variables. Pearson correlation coefficient was calculated to determine the association between two normally distributed continuous variables. Correlation between dichotomous categorical variable, gender, and other continuous variable was analyzed using point-biserial correlation coefficient. The software used for data analysis was JAMOVI (version 2.3.28) accessed on 7th march 2024 to test the meditational hypothesis and to conduct the path analysis.

## Results:

A significant positive correlation was observed in the following variables: barriers/gender (correlation coefficient = .212, P = .034) (Table 1). However, no association was found among gender, age, other domains of courage and oral health belief (P >.05)(Table 1). There was positive correlation between perseverance and nutritional preferences (correlation coefficient = .239, P =.017); courage and nutritional preferences (correlation coefficient = .241, P = .016) (Table 2). No association existed in the following parameters: age/gender, age/courage and its domains, gender/ courage and its domains, age/oral health behaviour and its domains, gender/ oral hygiene habits, oral hygiene habits/courage and its domains. (P >.05) (Table 2).

## Discussion:

Emotional experiences may act as heuristics in cognitive processing of information and thereby influencing beliefs [15, 16]. Cognitive interpretation of knowledge and information is conditioned by individual's beliefs [17]. It may be attributed to motivated reasoning. Personal experiences of invasive nature of dentistry and the associated emotions anxiety may have its impact over translation of oral health beliefs into oral health behaviours. Our actions and behaviours amidst emotions of fear in our day-to-day activities are influenced by the application of the virtue of courage. The conceptual path of impact of construct of health beliefs over oral hygiene habits in the present study demonstrates no significant direct or indirect impact (mediated through virtue of courage) in our study. This trend is reflected through the correlation among these parameters also. Cross-sectional design of the present study does not establish the direction of this impact. Patient compliance,[18,19] an important facet of health behaviours, is crucial to the successful management of diseases. However, compliance rate is reported to be low, particularly in preventive healthcare behaviours and engagements. Patient's beliefs about the disease and its management are considered to be important determinants in it. Individual characteristics might affect the individual's perceptions and might contribute significantly in influencing health related behaviours. The scope of the virtue of courage or the associated character strengths influencing perception about importance of oral health warrants further exploration. An interesting finding of the study is the negative correlation between 'perceived barriers to availing oral health' and oral hygiene habits. Items related to ‘perceived barriers to availing oral healthcare' in the questionnaire included the fear of dental visits and availability of dentists. Individuals not afraid of dental visits were observed to be impacted negatively with reference to oral hygiene habits. This finding of the study is contrary to the anticipated results of positive correlation of oral health beliefs and oral hygiene habits. In the conceptual mediated path, it is revealed to be the direct influence. Fear appeals are routinely observed to be employed for augmenting health behaviours. The scope of fear appeals in perceived pain and adverse feelings about invasive nature of dental treatment modulating oral hygiene habits positively in the study population cannot be ruled out. Positive correlation of perceived benefits of oral hygiene habits and motivation of visiting dentist for dental problems with oral hygiene habits further hints towards fear appeals in adverse feelings for invasive nature of dental treatment influencing primary preventive oral hygiene care positively to avoid adverse feelings with dental visits. Another interesting aspect of these findings about the study population is that inspite of perceived dental anxiety reflected through low score of items of perceived barriers to oral health action of dental visits; perceived benefits of oral hygiene habits bear direct positive impact over oral hygiene habits.

Perseverance character strength of psychological virtue of courage is observed to have significant positive impact over dietary habits conductive to dental health in the present study. As oral health beliefs are not found to be associated with character strength perseverance, hence mediated impact of oral health beliefs over dietary habits gets diluted to insignificant level. While impact of dietary habits over oral health, mediated through influence over oral micro biota, is well known; microbial constituents of dental plaque have also been reported to influence taste perception and dietary preferences for their survival [20, 21, 22-23]. This vicious cycle makes the role of dietary habits more relevant for oral health. Participants with low courage are observed to bear positive correlation of oral health benefits with oral health behaviours. However, this correlation is negative in participants with high courage. It may be attributed to effectiveness of fear appeals in the adverse feelings of invasive nature of dental treatment in participants with low courage. Aristotle conceptualized courage23 in terms of impact of fear on person's decisions. Relevance of decision making cannot be underestimated for normal functioning of individuals in day-to-day activities. Appreciation of risk and probable consequences may be an important element of decision making in health behaviours. Risk of facing invasive nature of dentistry may be driving force for augmenting positive oral behaviours in the population of the present study. Self-regulation during decision making has been reported to be associated with the virtue of courage. Patterns of thinking and behaving are reported to be driven by largely stable personality traits even during situations when erratic health behaviour may prove to be fatal [24]. The virtue of courage may be cultivated. Hence learning to be courageous may be a lifelong process. Individuals may fail in their responsibility to care for their health during non-courageous mode of functioning. Courageous coping may act as a potential mediator between resilience and social support, and between resilience and hope [25]. Cognitive appraisal of dental fear and anxiety has also been reported to facilitate implementation of oral health behaviour [26].

## Conclusion:

Character strengths of virtue of courage are found to have significant positive impact over dietary preferences compatible with oral health. It further hints towards the potential impact of virtue of courage related character strengths over health beliefs. However direction of relationship can't be established on the basis of this cross-sectional study. Clinical Relevance: perspective on human behaviour restricted to psychological weakness of pathological anxiety may hinder with balanced individual appraisal. Extraction and utilization of individual character strengths may not only be conductive to positive mental health but to oral health as well.

## Figures and Tables

**Table 1:** Correlation among demographic variables, domains of oral health belief, and domains of courage

**VARIABLES**		**Age**	**Gender**	**Bravery**	**Perseverance**	**Honesty**	**Zest**	**Courage**
Age	Correlation coefficient	1	0.095	0.06	0.123	0.026	0.109	0.089
	P value	-	.345^#^	.554^+^	.222^+^	.799^+^	.280^+^	.377^+^
Gender	Correlation coefficient	0.095	1	0.103	-0.002	0.016	-0.047	0.024
	P value	.345^#^	-	.306^#^	.984^#^	.872^#^	.642^#^	.811^#^
Perceived seriousness	Correlation coefficient	0.022	0.092	-0.01	0.018	-0.02	-0.018	-0.009
	P value	.828^+^	.362^#^	.918^+^	.861^+^	.842^+^	.856^+^	.930^+^
Barriers	Correlation coefficient	0.111	0.212	0.027	-0.018	-0.018	-0.069	-0.022
	P value	.273^+^	.034^#^*	.789^+^	.862^+^	.856^+^	.496^+^	.832^+^
Motivation	Correlation coefficient	0.194	0.118	0.045	0.013	-0.063	-0.106	-0.023
	P value	.053^+^	.244^#^	.657^+^	.898^+^	.536^+^	.293^+^	.820^+^
Perceived benefits	Correlation coefficient	0.028	-0.089	0.14	-0.028	-0.026	-0.004	0.044
	P value	.778^+^	.377^#^	.166^+^	.782^+^	.801^+^	.972^+^	.667^+^
Effectiveness	Correlation coefficient	0.164	0.101	0.096	0.122	0.084	0.014	0.098
	P value	.103^+^	.320^#^	342^+^	.226^+^	.407^+^	.890^+^	.334^+^
Oral health belief total	Correlation coefficient	0.088	0.065	0.163	0.004	-0.024	-0.023	0.044
	P value	.386^+^	.520^#^	.104^+^	.966^+^	.816^+^	.822^+^	.663^+^
*Statistical significance (P < .05)
^#^ Point-biserial correlation coefficient
^+^Spearman rank correlation coefficient

**Table 2:** Correlation among demographic variables, oral hygiene habits, and domains of courage

**VARIABLES**		**Age**	**Gender**	**Bravery**	**Perseverance**	**Honesty**	**Zest**	**Courage**
Age	Correlation coefficient	1	0.095	0.06	0.123	0.026	0.109	0.089
	P value	-	.345^#^	.554^+^	.222^+^	.799^+^	.280^+^	.377^+^
Gender	Correlation coefficient	0.095	1	0.103	-0.002	0.016	-0.047	0.024
	P value	.345^#^	-	.306^#^	.984^#^	.872^#^	.642^#^	.811^#^
Oral hygiene habits	Correlation coefficient	-0.149	-0.006	-0.037	-0.062	-0.066	-0.025	-0.06
	P value	.138^+^	.955^#^	.717^+^	.541^+^	.514^+^	.803^+^	.554^+^
Nutritional preferences	Correlation coefficient	-0.136	0.039	0.19	0.239	0.194	0.129	0.241
	P value	.176^+^	.701^#^	.058^+^	.017^+^*	.053^+^	.201^+^	.016^+^*
Statistical significance (P < .05);
^#^ Point-biserial correlation coefficient;
^+^Spearman rank correlation coefficient;
*Pearson correlation coefficient

**Table 3:** Mediation path-estimate of Barriers ⇒ Bravery ⇒ Oral hygiene habits

**Indirect and Total Effects**							
		**95% C.I. (a)**						
**Type**	**Effect**	**Estimate**	**SE**	**Lower**	**Upper**	**β**	**z**	**P**
Indirect	Barriers ⇒ Bravery ⇒ Oral hygiene habits	8.18E-04	0.00768	-0.0142	0.0159	2.18E-04	0.107	0.915
Component	Barriers ⇒ Bravery	0.017	0.11304	-0.2046	0.2385	0.015	0.15	0.881
	Bravery ⇒ Oral hygiene habits	0.0482	0.31895	-0.5769	0.6733	0.0146	0.151	0.88
Direct	Barriers ⇒ Oral hygiene habits	-1.0116	0.36057	-1.7184	-0.3049	-0.2702	-2.806	0.005
Total	Barriers ⇒ Oral hygiene habits	-1.0108	0.36239	-1.7211	-0.3006	-0.2699	-2.789	0.005
Note. Confidence intervals computed with method: Standard (Delta method)								

**Table 4:** Mediation path-estimate of Barriers ⇒ Perseverance ⇒ Oral hygiene habits

**Indirect and Total Effects**								
		**95% C.I. (a)**						
**Type**	**Effect**	**Estimate**	**SE**	**Lower**	**Upper**	**β**	**z**	**P**
Indirect	Barriers ⇒ Perseverance ⇒ Oral hygiene habits	0.00114	0.00945	-0.0174	0.0197	3.05E-04	0.121	0.904
Component	Barriers ⇒ Perseverance	-0.02292	0.10507	-0.2289	0.183	-0.0218	-0.218	0.827
	Perseverance ⇒ Oral hygiene habits	-0.0498	0.34312	-0.7223	0.6227	-0.014	-0.145	0.885
Direct	Barriers ⇒ Oral hygiene habits	-1.01197	0.36062	-1.7188	-0.3052	-0.2702	-2.806	0.005
Total	Barriers ⇒ Oral hygiene habits	-1.01083	0.36239	-1.7211	-0.3006	-0.2699	-2.789	0.005
Note. Confidence intervals computed with method: Standard (Delta method)								

**Table 5:** Mediation path-estimate of Barriers ⇒ Zest ⇒ Oral hygiene habits

**Indirect and Total Effects**								
		**95% C.I. (a)**						
**Type**	**Effect**	**Estimate**	**SE**	**Lower**	**Upper**	**β**	**z**	**P**
Indirect	Barriers ⇒ Zest ⇒ Oral hygiene habits	0.00187	0.0132	-0.024	0.0278	5.00E-04	0.142	0.887
Component	Barriers ⇒ Zest	-0.03141	0.0948	-0.2172	0.1544	-0.0331	-0.331	0.74
	Zest ⇒ Oral hygiene habits	-0.05966	0.3802	-0.8049	0.6856	-0.0151	-0.157	0.875
Direct	Barriers ⇒ Oral hygiene habits	-1.0127	0.3607	-1.7197	-0.3057	-0.2704	-2.807	0.005
Total	Barriers ⇒ Oral hygiene habits	-1.01083	0.3624	-1.7211	-0.3006	-0.2699	-2.789	0.005
Note. Confidence intervals computed with method: Standard (Delta method)								
